# Fragmented Adipose Tissue Graft for Bone Healing: Histological and Histometric Study in Rabbits’ Calvaria

**DOI:** 10.4317/medoral.18407

**Published:** 2013-03-25

**Authors:** Lidiane C. Oliveira, Allan F. Giovanini, Allan Abuabara, Luiz G. Klug, Carla C. Gonzaga, João C. Zielak, Cícero A. Urban, Tatiana M. Deliberador

**Affiliations:** 1 MSc. Master Program in Clinical Dentistry, Positivo University, Curitiba/PR, Brazil; 2 PhD. Master Program in Clinical Dentistry, Positivo University, Curitiba/PR, Brazil; 3 DDS. Oral and Maxillofacial Radiologist, Joinville Municipal Authority, Joinville, Santa Catarina, Brazil

## Abstract

Objective The adipose tissue represents an important reservoir of stem cells. There are few studies in the literature with which to histologically evaluate whether or not the adipose tissue graft is really a safe option to achieve bone repair. This study histologically analyzed the effect of fragmented autogenous adipose tissue grafts on bone healing in surgically created, critical-size defects (CSD) in a rabbit’s calvaria. Study design Forty-two New Zealand rabbits were used in this study. CSD that were 15 mm in diameter were created in the calvarium of each animal. The defects were randomly divided into two groups: in Group C (control), the defect was filled only by a blood clot and, in Group FAT (i.e., fragmented adipose tissue), the defect was filled with fragmented autogenous adipose tissue grafts. The groups were divided into subgroups (n = 7) for euthanasia at 7, 15, and 40 days after the procedure had been conducted. Histologic and histometric analyses were performed. Data were statistically analysed with ANOVA and Tukey’s tests (p < 0.05). Results The amount of bone formation did not show statistically significant differences seven days after the operation, which indicates that the groups had similar amounts of mineral deposition in the earlier period of the repair. Conversely, a significant of amount of bone matrix deposition was identified in the FAT group at 15 and 40 days following the operation, both on the border and in the body of the defect. Such an outcome was not found in the control group. Conclusion In this study, an autologous adipose tissue graft may be considered as likely biomaterial for bone regeneration, since it positively affected the amount of bone formation in surgically created CSD in the rabbits’ calvaria 40 days after the procedure had been performed. Further investigations with a longer time evaluation are warranted to determine the effectiveness of autologous adipose tissue graft in the bone healing.

** Key words:**Adipose tissue, bone regeneration, rabbits, critical defects.

## Introduction

A challenge in plastic and reconstructive surgery is to replace tissue and restore function through the transfer of tissue from other parts of the body (1). Bone replacement is often necessary during the reconstruction of craniofacial anomalies or trauma (2). When reconstructing defects in bone, current options for tissue coverage and restoration include autologous bone grafts, cadaveric bone grafts, pedicle or free-tissue transfer, and allotransplantation (3-7). The repair of large skull defects may be difficult, due to the limited amount of autogenous bone that is available (8). Due to its limited availability, morbidity of the autogenous bone graft, and an inadequate potential osteoinductive and osteoconductive of the allograft, research has been conducted to develop new strategies to improve bone healing.

Adipose-derived stem cells have been identified as a source of multipotent cells that have osteogenic differentiation potential in vitro and in vivo (2,9). With the discovery of pluripotent adipose-derived stem cells, tissue engineering may offer a viable alterna-tive (1,10). In 2001, Zuk et al. (11) demonstrated the capacity of adipose-derived stem cells for in vitro differentiation into several mesodermal lineages, including fat, bone, and cartilage. This group also characterized the expression profile of several osteogenic transcripts and proteins in the osteoinduced adipose-derived stem cells (12). Lee et al. (13) demonstrated in vivo bone formation by using osteoinduced human adipose-derived stem cells that were seeded onto polylactic acid/polyglycolic acid (PLA/PGA) scaffolds and placed in subcutaneous pockets in severe combined immunodeficient mice. Lendeckel et al. (8) first reported the use of adipose-derived stem cells to augment cancellous bone for the treatment of a difficult reconstructive calvarial defect.

Adipose tissue is derived from embryonic mesenchymal cells and contains a stromal structure that is similar to bone marrow stem cells (7). Adipose-derived stem cells are obtained without the morbidity of bone marrow harvesting and have been induced to express genes and protein markers that are associated with osteoblasts, chondrocytes, adipocytes, endothelium, and myocytes (11,12,14,15). The incorporation of autogenous adipose-derived stem cells into allograft tissues can easily be translated into the current clinical practice by using banked cadaveric tissues (7). Stem cell frequency is significantly higher in adipose tissue in comparison to bone marrow (2% vs. 0.002%) (16).

Despite recent advances in the use of adipose-derived stem cells, the use of adipose tissue graft has not yet been evaluated in vivo as an alternative treatment for bone repair. The aim of this study was to histologically analyze the effect of fragmented autogenous adipose tissue grafts on bone healing in surgically created, critical-size defects (CSD) in the rabbits’ calvaria.

## Material and Methods

-Experimental Model

The Ethics and Research Committee from Positivo University, Curitiba/PR, Brazil, approved this study protocol. All guidelines regarding the care of animal research subjects were strictly followed.

Forty-two male New Zealand white rabbits (Oryctolagus cuniculus) that weighed approximately 3.000 ± 0.550kg were used for this study. This species of rabbit was selected because of its small size, simple acquisition, reasonable cost, and convenient care in the laboratory (17). Furthermore, the unique anatomic structure of these rabbits presents a great advantage. They have a large adipose tissue pouch with a definite location and easy access (17).

The animals were randomly assigned to one of two experimental groups: Group C (control) and Group FAT (fragmented adipose tissue).

-Surgical Procedure

Twenty-four hours before the surgical procedure, the animals received an intramuscular (i.m.) injection of enrofloxacin (0.1 ml/kg of body weight). The rabbits were sedated by an i.m. injection of midazolam (1 mg/kg) and were anaesthetized by an i.m. injection of xylazine (5mg/kg) and ketamine (35 mg/kg).

After an aseptic preparation, a V-shaped incision was made in the skin over the top of the cranial vault and a cutaneous flap was raised and reflected in a posterior direction to expose the periosteum. In the periosteum, a similar incision was made, followed by a gentle subperiosteal dissection. A transosseous CSD (15 mm in diameter) was produced with a trephine under continuous sterile saline irrigation. The defect included a portion of the sagittal suture.

**Figure 1:**
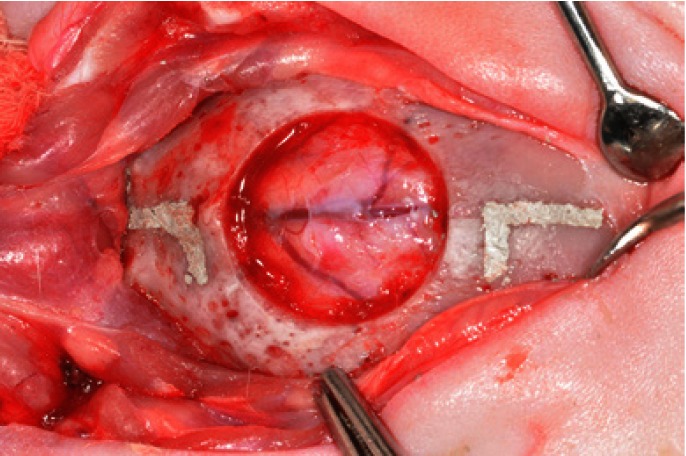
Critical-size defect (15 mm diameter) and the two reference marks created on the calvarium.

Two L-shaped marks were produced (i.e., 2 mm anterior and 2 mm posterior to the margins of the artificial surgical defect) by using a small tapered carbide fissure bur and a surgical stent. The long axes of the L-shaped marks were located on the longitudinal axis bisecting the surgical defect. The marks were filled with amalgam (Fig. 1) for posterior identification of the center of the original defect during laboratory processing and as references to accurately locate the original bone margins of the surgical defect during histometric analysis (18).

**Figure 2:**
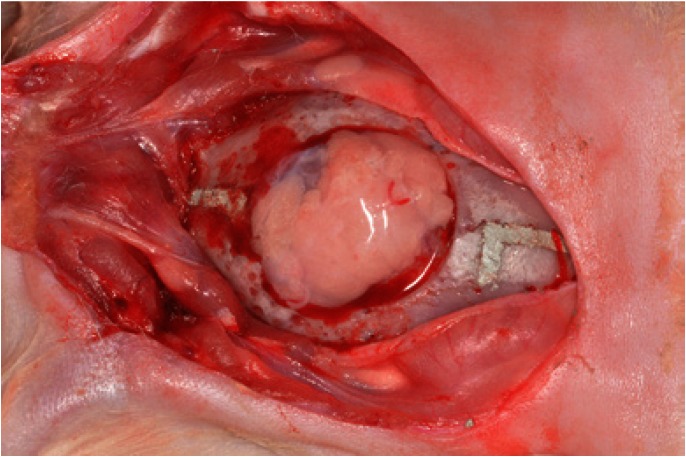
Autologous adipose tissue graft filling the critical-size defect.

In Group C, the defect was filled only by a blood clot. In Group FAT, the defect was filled with 86 mm3 of fragmented autogenous adipose tissue grafts that were removed from the hypoderms of the rabbit (Fig. 2). The soft tissues were repositioned and sutured to achieve primary closure. The periosteum was repositioned and sutured with absorbable sutures (5-0 Vicryl) and the skin was sutured with non-absorbable sutures (4-0 Silk).

Post-surgically, each animal received daily i.m. injections of enrofloxacin (5 ml/kg) for 5 days. For control of postoperative pain, the animals received i.m. injections of morphine (3 mg/kg) at the conclusion of the surgery and 4 hours after the first application and acetaminophen (200 mg/kg) 3 times daily for 5 days.

-Euthanasia Procedure and Tissue Processing

Each group of animals was divided into three subgroups (n = 7) for euthanasia at either 7, 15 and 40 days following the operation. After general anesthesia by i.m. injections of xylazine (5 mg/kg) and ketamine (35 mg/kg), the animals were euthanized with an overdose of intravenous sodium thiopental (10 mg/kg). The area of the original surgical defect and the surrounding tissues were removed en bloc.

The specimens were fixed in 10% buffered formalin for 48h and decalcified in 20% formic acid and sodium citrate for 10 days. After the initial decalcification, each specimen was divided longitudinally into two blocks exactly along the centre line of the original surgical defect by using the long axis of both L marks as references. Transverse cuts were then made by using the short axis of each L mark as a reference. Each specimen then measured 19 mm in length along the longitudinal axis that ran through the centre of the defect, allowing for identification of the original surgical defect margins during both histologic and histometric evaluations (19).

After complete decalcification, the specimens were washed with tap water, dehydrated, diafanized, and embedded in paraffin. Serial sections of 3 ?m parallel to the mid-sagittal suture (longitudinal direction) were cut from the center of each defect by using a microtome. They were also stained with hematoxylin and eosin (H.E.) for analysis by light microscopy.

-Histomorphometric Analysis

Two histologic sections that represented the centre of the original surgical defect were selected. The histologic and histometric analyses were performed by an examiner who was blinded with respect to the treatment that had been rendered.

The images of the histological sections were captured by a digital camera coupled with a light microscope with an original magnification of ×40. Each digital image was saved with 600 dpi resolution, producing a virtual frame of 117 × 80 cm. Because it was not possible to capture the entire defect in one image at the magnification level that was used, a composite digital image of the whole defect was then constructed by combining eight smaller images based on histomorphological reference structures, especially the deposited bone trabeculae and blood vessels.

All histomorphometric measurements were completed using the software Image Tool 3.0 (IT – UTHSCSA (The University of Texas Health Science Center, San Antonio, TX, USA). An image of a 1-mm slide was used to calibrate all measurements. The histomorphometric data were counted manually and expressed as areas (in mm2).

The following criteria, based in part on the work of Messora et al. (18) and (19), were used to standardize the histomorphometric analysis of the digital images:

1- The total area (TA) to be analyzed corresponded to the entire area of the original surgical defect. This area was determined by first identifying the external and internal surfaces of the original calvarium at the right and left margins of the surgical defect, and then connecting them with lines drawn following their respective curvatures. The center of the histological section (considering its total length) was localized and 2mm were measured to the right and to the left of this center point in order to determine the limits of the original surgical defect.

2- The mineral deposition area (MDA) was delineated within the confines of the TA.

3- The TA was measured in mm2 and was considered 100% of the area to be analyzed. The MDA was also measured in mm2 and calculated as a percentage of TA.

The descriptive histological evaluation assessed the closure of the defect, the type of newly formed bone, the characteristics of the connective tissue, the presence of osteoid matrix, and the remnants of the adipose tissue graft, as well as the type of inflammatory infiltrate. The closure of the defect was considered complete when all of its length was filled with new bone/mineral deposition.

-Statistical Analysis

The values of MDA for each animal were represented by the mean percentage of the two histological sections. Data were statistically analyzed using ANOVA and Tukey’s tests with a significance level of 5%.

## Results

All animals tolerated the surgical procedures well. Healing was uneventful in groups having a similar clinical presentation.

-Histological Analysis 

Group C 

**Figure 3:**
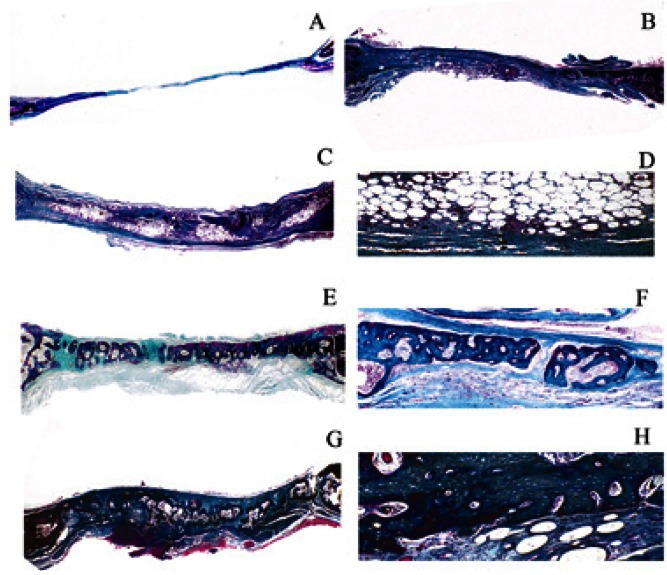
Microscopic images of the groups. Micrograph A) and B) reveal the histological features in Group C at 15 and 40 days post surgery, respectively (Masson’s trichrome, Original magnification × 12). Verify that the biggest part of the surgical defect was occupied by fibrous connective tissue in the groups post-surgery, while the bone matrix deposition was scarce and restricted to the border of the defect. Micrograph C) and D) reveals the histological features in Group FAT at 15 days post-surgery (Masson’s trichrome, Original magnification × 12 and × 40, respectively). The images demonstrate the presence of connective tissue surrounding the areas occupied by remaining adipose tissue that was grafted. Micrograph E) and F) reveals the histological features in Group FAT at 40 days post-surgery (Masson’s trichrome, Original magnification × 12 and × 40, respectively). Shows areas composed by an osteoid matrix and isolated fragments of mature new bone formation. Micrograph G) and H) reveals the histological features in Group FAT at 40 days post-surgery (Masson’s trichrome, Original magnification × 12 and × 40, respectively). Verify that the biggest part of the defect was composed by a Haversian mature bone matrix.

The complete closure of the defect was not observed at 7, 15 (Fig. 3), and 40 days (Fig. 3). Newly formed bone was present yet restricted to areas that were close to the borders of the surgical defect. The biggest part of the surgical defect was occupied by connective tissue with collagen fibers parallel to the wound surface (Fig. 3) with a mild to moderate chronic inflammatory infiltrate. At 40 days, there was a mild chronic inflammatory process and large amounts of collagen fibers arranged parallel to the wound surface. However, new bone fragments were identified only in the margins of the surgical defect (Fig. 3).

Group FAT 

The complete closure of the defect was not observed at the 7–day mark. The biggest part of the surgical defect was occupied by the remaining adipose tissue graft which was surrounded by fibrous connective tissue. Newly formed bone was restricted to areas close to the borders of the surgical defect. After 15 days, the histological features demonstrated a significant increase of new bone tissue that was deposited both in the margins and the connective caps that surrounded the adipose tissue. (Fig. 3). After 40 days, complete closure of the defects had not yet been verified in all specimens. However, the rabbits treated with FAT showed areas composed by an osteoid matrix and isolated fragments of mature new bone formation (Fig. 3). In some specimens was verified the presence of Haversian mature bone matrix (Fig. 3).

-Histometric and Statistical Analyses

The data normality and homogeneity of variances were verified. Means and standard deviations of MDA for two groups, as well as the comparison between the groups, at 7, 15, and 40 days post-operation are documented in  Table 1. The amount of bone formation did not show any statistically significant differences in the 7-day post-operative period, which indicates that the groups had similar amounts of mineral deposition in the earlier period of the repair. However, a significant amount of bone matrix deposition was identified in the FAT group at 15 and 40 days post-operation, both on the border and in the body of defect. Such facts were not found in the control group.

**Table 1:**
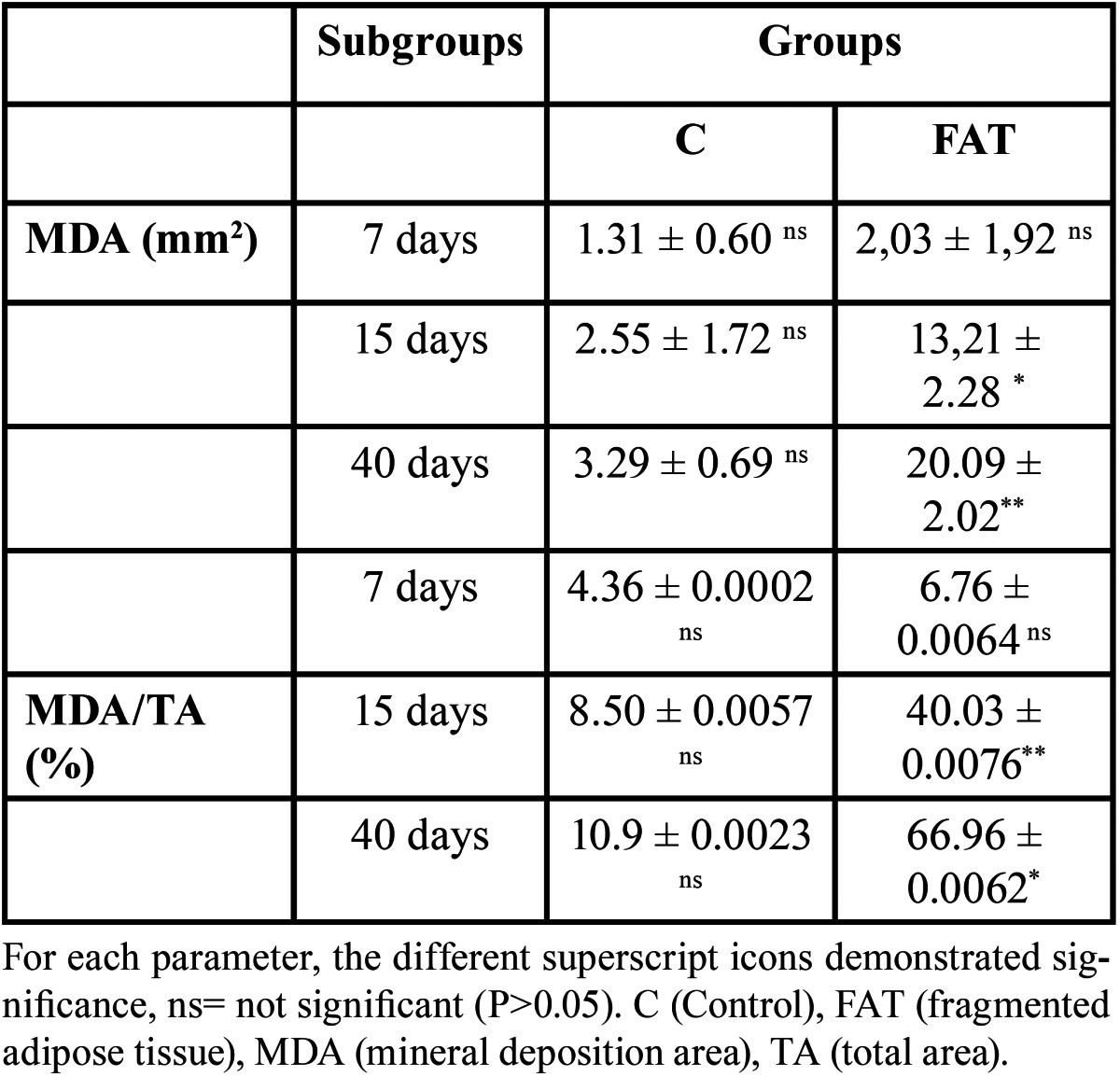
Means and standard deviations of mineral deposition area (MDA) and mean percentage of MDA within the total area (TA) of the surgically created defect. Comparison between the groups at 7, 15 and 40 days post-operative.

## Discussion

It is known that the mesenchymal stem cells residing in bone marrow can be differentiated into osteoblasts, chondrocytes, adipocytes, and muscle cells under given conditions in vitro (20). However, bone-marrow-derived mesenchymal stem cells are not the only stem cells that can differentiate into various skeletal tissues. Fat-derived stromal cells also possess similar properties (21,22). Since 2001, adipose tissue has been found to represent a convenient cell source for tissue engineering and regenerative medicine, mainly due to its abundance and ease of harvesting, with minimal associated morbidity as well as a much higher frequency of clonogenic mesenchymal progenitors compared to bone marrow (11,23,24). In addition to these features, it has been recognized that the non-adipocyte fraction of adipose tissue is also rich in endothelial cells and progenitors, suggesting that it could represent a unique and abundant source of autologous osteogenic and angiogenic progenitors (24).

This study histologically analyzed the effect of fragmented autogenous adipose tissue grafts on bone healing in surgically created critical-size defects (CSD) in the rabbits’ calvaria. We believe that this study is the first that has evaluated the use of adipose tissue graft to repair calvarial bone defects in rabbits. Some studies with rats (7), rabbits (2), and dogs (25) evaluated the bone formation by using stromal stem cells that were derived from adipose tissue, but not with an adipose tissue graft. These studies (2,7,25) concluded that stromal stem cells that are derived from adipose tissue have the potential for osteogenic differentiation.

In a pilot study with rabbit’s tibiae, Godoy Zanicotti et al. (26) evaluated non-processed adipose tissue graft in the treatment of peri-implant osseous defects. According to the authors, the use of non-processed adipose tissue around surgically created peri-implant osseous defects interfered negatively with the bone formation (26). The adipose tissue seems to impede the bone formation around the implant. Contrary to Godoy Zanicotti et al. (26), the results of the present study showed new bone formation in the treatment group (i.e., fragmented adipose tissue graft).

The data found in this study showed maturation of granulation tissue, several areas with an osteoid matrix, and significant deposition of compact mature bone tissue 15 days post surgery. In addition, several areas of adipose tissue still were present in Group FAT at 7 and 15 days. Although also it was observed in Group FAT at the 40–day mark in small areas with remnant adipose tissue, the specimens of this group presented significant areas of both osteoid matrix and of Haversian-compact bone tissue formation. None of the surgical defects in any of the groups were completely repaired with bone.

It is noteworthy that, during the healing process, we observed that the adipose tissue was absorbed and replaced by bone tissue, but neither dehiscence or loss of tissue volume of the grafted area was identified histologically. For this reason, we addressed the possibility of adipose cell transdifferentiation into osteoblasts, although this study has not evaluated the specific origin of the cells that formed bone. Transdifferentiation is usually defined as the irreversible switch of one type of differentiated cell to another. It belongs to a wider class of cell type transformations called metaplasias, which also includes cases in which stem cells of one tissue type switch to a completely different stem cell, but maintaining the same embryonic characteristic of the original tissue (27). Normally dedifferentiation and cell division are essential intermediate processes in the switch in phenotype, but may not be obligatory in all cases. Transdifferentiation is associated with a discrete change in the programme of gene expression and there is a direct ancestor-descendant relationship between the two cell types (27). At the molecular level, the cause of transdifferentiation is presumably a change in the expression of a master switch gene (i.e., a selector or homeotic gene), whose normal function is to distinguish the two cell types in normal development (27). Numerous examples of transdifferentiation exist within the literature (27,28).

In vivo and in vitro studies have demonstrated the capacity of differentiation of mature adypocites into bone tissue (29). Zou et al. (30) showed that adipose-derived stromal cells have the potential to differentiate into osteogenic lineage, both in vitro and in vivo. In agreement with Zou et al. (30), we believe that adipose tissue can be a promising novel cell-based therapy for healing bone defects in the clinical setting. In this study, even using only adipose tissue graft to repair bone defects rather than stem cells from fat tissue, we observed that the adipose tissue graft has osteogenic potential. Such an outcome was evident in Group FAT 40 days after the procedure. Stem cells are present in adipose tissue at a frequency of 2% and it has been identified as having differentiation potential that extends beyond the osteogenic phenotype. Reports have indicated differentiation in vitro and in vivo towards adipogenic, chondrogenic, myogenic, neurogenic, endothelial, hematopoietic, and cardiomyogenic phenotypes (16).

The use of the adipose tissue graft simulated a clinical situation for the repair of bone defects without the laboratory processing of stem cells. Thus, we can reduce treatment costs and time. Adipose tissue might have advantages over bone marrow, such as: (i) minimal morbidity upon harvest; (ii) clinically relevant stem cell numbers that are extractable from tissue isolates, potentially removing the need for in vitro propagation; (iii) stem cell frequency is significantly higher in adipose tissue compared with bone marrow (2% vs. 0.002%); and (iv) higher proliferation rates than bone marrow stem cells (16). In the FAT group 40 days following the operation, several areas of bone formation were present. This finding suggests the important potential of this tissue in haversian bone tissue engineering.

## Conclusion

Within the limits of the present study, we concluded that an autologous adipose tissue graft can positively affect the amount of bone formation in surgically created CSD in the rabbits’ calvaria 40 days following the procedure. Further investigations with a longer time evaluation are warranted to determine the effectiveness of autologous adipose tissue graft in the bone healing.
